# Metabolomic Profiling Reveals Differences in Hypoxia Response between Far Eastern and Siberian Frogs

**DOI:** 10.3390/ani13213349

**Published:** 2023-10-27

**Authors:** Sergei V. Shekhovtsov, Nina A. Bulakhova, Yuri P. Tsentalovich, Ekaterina A. Zelentsova, Nataliya A. Osik, Ekaterina N. Meshcheryakova, Tatiana V. Poluboyarova, Daniil I. Berman

**Affiliations:** 1 Institute of Cytology and Genetics SB RAS, Lavrentieva av. 10, 630090 Novosibirsk, Russia; tanita11@mail.ru; 2 Institute of the Biological Problems of the North FEB RAS, Portovaya 18, 685000 Magadan, Russia; sigma44@mail.ru (N.A.B.); katusha@ibpn.ru (E.N.M.); dberman@mail.ru (D.I.B.); 3 International Tomography Center SB RAS, Institutskaya 3a, 630090 Novosibirsk, Russia; yura@tomo.nsc.ru (Y.P.T.); zelentsova@tomo.nsc.ru (E.A.Z.); n.osik@tomo.nsc.ru (N.A.O.)

**Keywords:** metabolomics, *Rana dybowskii*, *Rana amurensis*, hypoxia

## Abstract

**Simple Summary:**

The lack of oxygen is a significant challenge for most animals, which can lead to tissue damage and death. The Siberian frog *Rana amurensis* is so far the only known amphibian capable of surviving water anoxia for a long time. We compared metabolomic profiles of the liver, brain, and heart under hypoxia for the Siberian frog and the Far Eastern (*Rana dybowskii*) frog, which is highly susceptible to the lack of oxygen. One of the most interesting findings was that the organs of the Far Eastern frog had more lactate (glycolysis end product) than those of the Siberian frog despite a much shorter exposure time. The amounts of succinate were similar between the two species. Interestingly, glycerol and 2,3-butanediol were found to be abundant under hypoxia in the Siberian frog, but not in the Far Eastern frog. The role of these substances are still unclear. Based on the obtained data, we suggest a bioenergetic pathway for metabolic changes in the Siberian frog under anoxia.

**Abstract:**

Anoxia is a significant challenge for most animals, as it can lead to tissue damage and death. Among amphibians, the Siberian frog *Rana amurensis* is the only known species capable of surviving near-zero levels of oxygen in water for a prolonged period. In this study, we aimed to compare metabolomic profiles of the liver, brain, and heart of the Siberian frog exposed to long-term oxygen deprivation (approximately 0.2 mg/L water) with those of the susceptible Far Eastern frog (*Rana dybowskii*) subjected to short-term hypoxia to the limits of its tolerance. One of the most pronounced features was that the organs of the Far Eastern frog contained more lactate than those of the Siberian frog despite a much shorter exposure time. The amounts of succinate were similar between the two species. Interestingly, glycerol and 2,3-butanediol were found to be significantly accumulated under hypoxia in the Siberian frog, but not in the Far Eastern frog. The role and biosynthesis of these substances are still unclear, but they are most likely formed in certain side pathways of glycolysis. Based on the obtained data, we suggest a pathway for metabolic changes in the Siberian frog under anoxia.

## 1. Introduction

Hypoxia poses a significant challenge to most terrestrial vertebrates. Mammals, even the most hypoxia-tolerant ones like the naked mole-rat [1,2], can survive anoxia only for minutes. Amphibians and reptiles are generally more resilient than mammals, mostly due to their lower metabolic rate [3]. However, species-specific adaptations also play a significant role in their survival [3]. For example, some turtle species can spend the winter underwater with almost no dissolved oxygen [4,5,6]. While even the most hypoxia-tolerant amphibians are known to survive only for a few days at low oxygen [3], recent studies identified two species capable of surviving extreme hypoxia. One is the Siberian frog *Rana amurensis* Boulenger, 1886 that demonstrates exceptional tolerance for this group [7]. This species can survive for several months under very low oxygen content at low positive temperatures while retaining the ability to move and react to stimuli. The other is the Pallas’ spadefoot *Pelobates vespertinus* (Pallas, 1771) that overwinters underground at 10% of the normal oxygen content [8]. The details of the physiological responses of the highly tolerant species to hypoxia are of particular interest.

Metabolomic analysis allows one to obtain data for dozens of metabolites simultaneously from a small tissue sample [9,10]. Although metabolomic studies on amphibians are still relatively scarce, some interesting species have already been investigated: the Xizang Plateau frog *Nanorana parkeri* (Stejneger, 1927) [11,12], the moor frog *Rana arvalis* Nilsson, 1842 [13], and the Siberian salamander *Salamandrella keyserlingii* Dybowski, 1870 [14]. In a previous study, we investigated the metabolomes of the heart and liver of the hypoxia-tolerant Siberian frog [15]. We found significant changes under exposure to hypoxia, including the accumulation of the products of glycolysis and several other biomolecules. However, the interpretation of these data is impeded by the lack of a reference species. To address this issue, here we attempted to add data for the Far Eastern frog *Rana dybowskii* Günther, 1876. This species is known to overwinter in streams with fast-flowing, oxygen-rich water. Its hibernation often occurs in bottom pits at the mouths of tributaries that flow into rivers, as well as in shallow sections of rivers with polynyas. The oxygen content in such places is high—ranging from 7 mg/L under the ice to 12–13 mg/L in shallow river sections without ice (our unpublished data). Laboratory experiments suggest that Far Eastern frogs are highly intolerant to hypoxia [16]. Conversely, the hypoxia-tolerant species *R. amurensis* mostly hibernates in stagnant water bodies, many of which experience severe winter freezing, with winter oxygen concentration dropping substantially below 1 mg/L (sometimes reaching anoxia). In order to perform such a comparison, we collected Far Eastern frogs during autumn and divided them into the control and the experimental samples. *R. dybowskii* is particularly vulnerable to hypoxia, and critical conditions can arise within hours of exposure, as opposed to the Siberian frog, which takes months. Once the individuals showed signs of critical conditions, we collected organs for metabolomic analysis. We used ^1^H-NMR analyses to quantify metabolites in the heart, liver, and brain as described in [15]. Comparing tolerant and susceptible species would allow us to elucidate the biochemical basis underlying the remarkable hypoxia tolerance exhibited by *R. amurensis* and could have significant practical applications. Additionally, we obtained data for *R. amurensis* brain metabolomes under hypoxia and normoxia. The brains of most species suffer irreversible damage under oxygen deprivation [3], so studying how it can remain viable in *R. amurensis* is of significant interest to the field.

## 2. Materials and Methods

### 2.1. Animal Maintenance

Siberian and Far Eastern frogs were captured on 22 September 2022, near the village of Arkhara in the Amur oblast (49° N, 130° E). The collected frogs were placed in 10 L open containers filled with water containing 5–7 mg/L of oxygen, 6 individuals in each container. The animals were kept for 2 days at 14–15 °C, followed by 4 days at 8 and 4 °C each, and then transferred to 2–3 °C. Throughout the entire captivity period, the oxygen concentration in the water for the amphibians was maintained in the range of 5–7 mg/L by changing the water every other day. The captured frogs were not fed since they do not feed during this season in the wild. Twenty days later, 12 individuals of each species were randomly assigned to control and experimental groups, with 6 individuals in each. The control (normoxic) groups remained in water with an oxygen content of 5–7 mg/L at a temperature of 2–3 °C. The frogs in the experimental group were transferred to experimental containers (with tightly sealed lids) filled with water containing 7 mg/L of oxygen at 2–3 °C. All 6 individuals of *R. amurensis* were placed in one container with a volume of 10.3 L, while each individual of *R. dybowskii* was placed in a separate container with a volume of 0.63 L. In the experiment, the oxygen concentration in the water decreased due to the animals’ respiration. The condition of the frogs (motor activity, level of behavioral disorders) was monitored by observing them through the transparent walls of the container.

In the container with *R. amurensis*, the oxygen content reached 0.1 mg/L after 7 days and remained at this level throughout the experiment. The dissolved oxygen content was measured using a digital single-channel multiparameter device, HACH HQ30D Flexi, with a luminescent sensor, LDO101 (Hach Lange, Düsseldorf, Germany). The instrument accuracy was 0.1 mg/L. The frogs were active during this time—they swam, moved around the bottom, or sat in normal positions without showing signs of depression. The animals were observed through the transparent walls of the container every 24 h. After 10 days in water with an oxygen concentration of 0.1 mg/L, the frogs were euthanized by rapid decapitation, and tissue samples were taken for analysis and frozen using liquid nitrogen. Animal handling was performed according to the directions of the Bioethics Committee of the Institute of Cytology and Genetics SB RAS (Protocol no. 153) within the budget project FWNR-2022-0022.

In the case of the experimental *R. dybowskii*, signs of depression and near-coma state appeared after only 1.5 days—the pupils were significantly constricted, body muscles were relaxed, and the animal hardly reacted to flipping the container or touching it with a thin plastic probe. Four hours after reaching this state, the frogs were euthanized, tissue samples were taken for analysis, and the oxygen content in water was measured. It ranged from 0.9 to 1.1 mg/L in different containers.

### 2.2. Metabolites Extraction

The preparation of the metabolomic extracts from Far Eastern frog liver, heart, and brain tissues, and from Siberian frog brain tissue was carried out according to the procedure described in [15,17]. Briefly, each weighted sample of the tissue was disintegrated with a TissueRuptor II homogenizer (Qiagen, Venlo, The Netherlands) in 1600 μL of cold methanol (−20 °C), and after that, 800 μL of water and 800 μL of cold chloroform were added to homogenate (−20 °C). Then the mixture was shaken with a shaker for 20 min and kept at −20 °C for 30 min. The centrifugation of the mixture (16,100× *g*, +4 °C, 30 min) yielded two immiscible liquid fractions separated by a protein layer. The upper (methanol–water) fraction was collected and lyophilized.

### 2.3. NMR Measurements

For NMR measurements, the lyophilized extracts were dissolved in D_2_O with 2 × 10^−5^ M sodium 4,4-dimethyl-4-silapentane-1-sulfonic acid (DSS, internal standard), and 20 mM deuterated phosphate buffer (pH 7.2). The ^1^H-NMR measurements were carried out in the Center of Collective Use «Mass spectrometric investigations» SB RAS on an AVANCE III HD 700 MHz machine (Bruker BioSpin, Ettlingen, Germany). ^1^H-NMR spectra were obtained with 64 accumulations of the free induction decay signal (with 90° detection pulse) with 12 s repetition time between scans. Water signal pre-saturation was performed by the use of low-power radiation at the water resonance frequency. Temperature of the sample during the data acquisition was kept at 25 °C. Metabolite concentrations in the samples were determined by integrating the peak areas relative to the internal standard.

### 2.4. Data Analysis

The baseline processing, identification, and integration of NMR spectra were performed using the MestReNova v12.0 (Mestrelab Research, Santiago, Spain). The attribution of spectral signals to metabolites and their quantification was performed as described in [15] with the use of the Human Metabolome Database [9] and our own database (https://amdb.online) (accessed on 8 August 2023). Only compounds with reliable identification and quantification were included in the data analysis. The criteria for data exclusion were: (a) unreliable metabolite identification: only one NMR signal was detected, and we did not have a standard compound to verify the identification; (b) unreliable quantification: the observed NMR signal was strongly overlapped by signals from other compounds. For further analysis, the data obtained in the present work were combined with the concentrations of metabolites in *R. amurensis* heart and liver obtained in the previous study [15].

The chemometric analysis of obtained data including principal component analysis (PCA) was performed on a MetaboAnalyst 5.0 web-platform (https://www.metaboanalyst.ca/) (accessed on 8 August 2023). PCA scores, loading plots, and volcano plots were constructed with the data auto-scaled (mean-centered and divided by the standard deviation of each metabolite concentration) to normalize the contributions of all metabolites. The parameters for the volcano plots are: FC > 1.5, *p* < 0.1, FDR-corrected.

## 3. Results

### 3.1. Metabolomes of the Heart and Liver of the Far Eastern Frog

A total of 62 substances were identified in the liver of the Far Eastern frog. The set of substances was similar in comparison to the Siberian frog, but the quantitative composition was significantly different even under normoxia (Figure S1A). A total of 19 of the detected substances showed statistically significant changes in response to hypoxia: 14 exhibited an increase in concentration, and 5, a decrease. The metabolites that showed the most pronounced accumulation under hypoxia were lactate (28-fold), alanine (9-fold), β-alanine (6-fold), glucose (5-fold), glycerol (4-fold), and succinate (3-fold).

**Figure 1:**
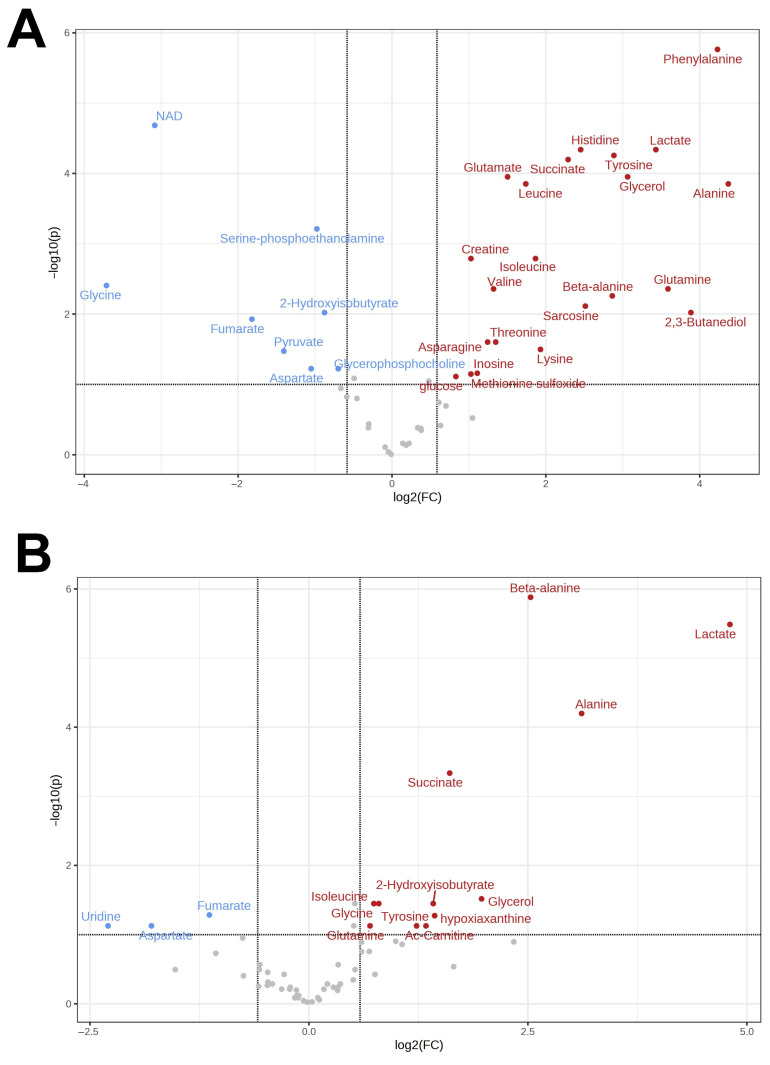
Volcano plots for the comparison of the metabolomic changes in the liver under hypoxia as compared to normoxia for *R. amurensis* (**A**) and *R. dybowskii* (**B**). Substances with increased concentrations under hypoxia are shown in red; decreased, in blue.

**Figure 2:**
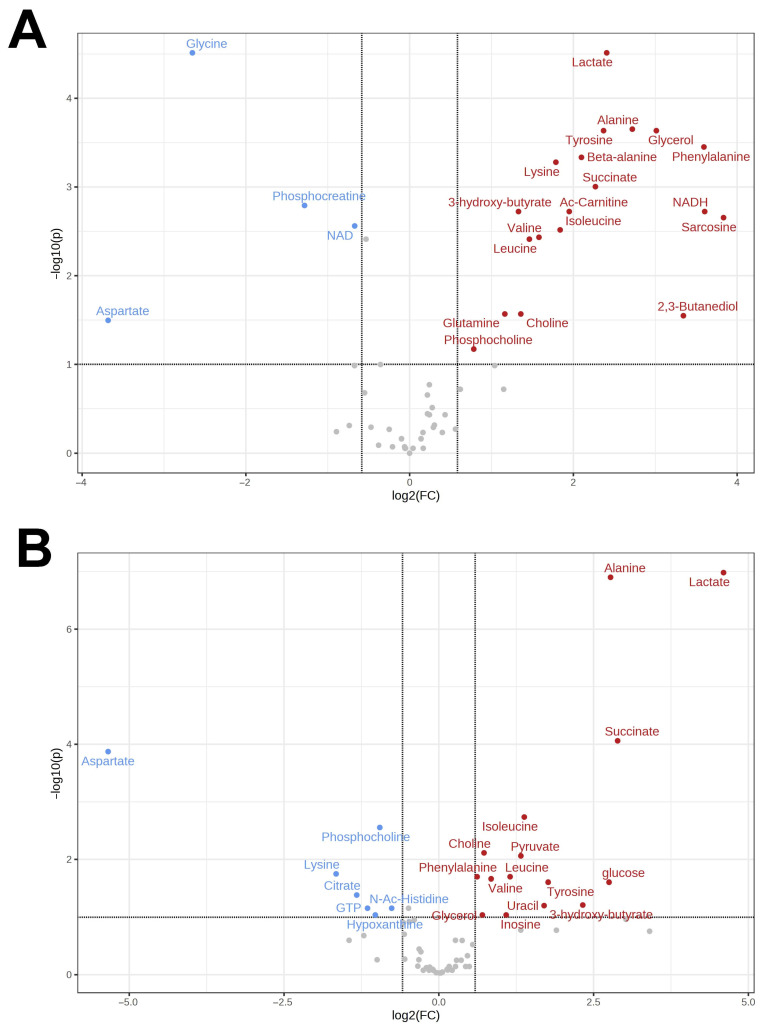
Volcano plots for the comparison of the metabolomic changes in the heart under hypoxia as compared to normoxia for *R. amurensis* (**A**) and *R. dybowskii* (**B**). Substances with increased concentrations under hypoxia are shown in red; decreased, in blue.

In the heart of the Far Eastern frog, 67 metabolites were quantified, with 15 of them demonstrating increased concentrations in response to hypoxia, and 7 showing a decrease. Again, the qualitative profiles differed from those of normoxic Siberian frogs (Figure S1B). The same molecules that exhibited significant changes in the liver were also found to have increased concentrations in the heart, including lactate (24-fold), alanine (7-fold), glucose (7-fold), and succinate (7.5-fold). In addition, there was a 40-fold decrease in aspartate content. The comparison of major metabolomic changes in tissues of Far Eastern and Siberian frogs is demonstrated in volcano plots (Figure 1 and Figure 2).

### 3.2. Brain Metabolomes of the Siberian and the Far Eastern Frogs

**Figure 3:**
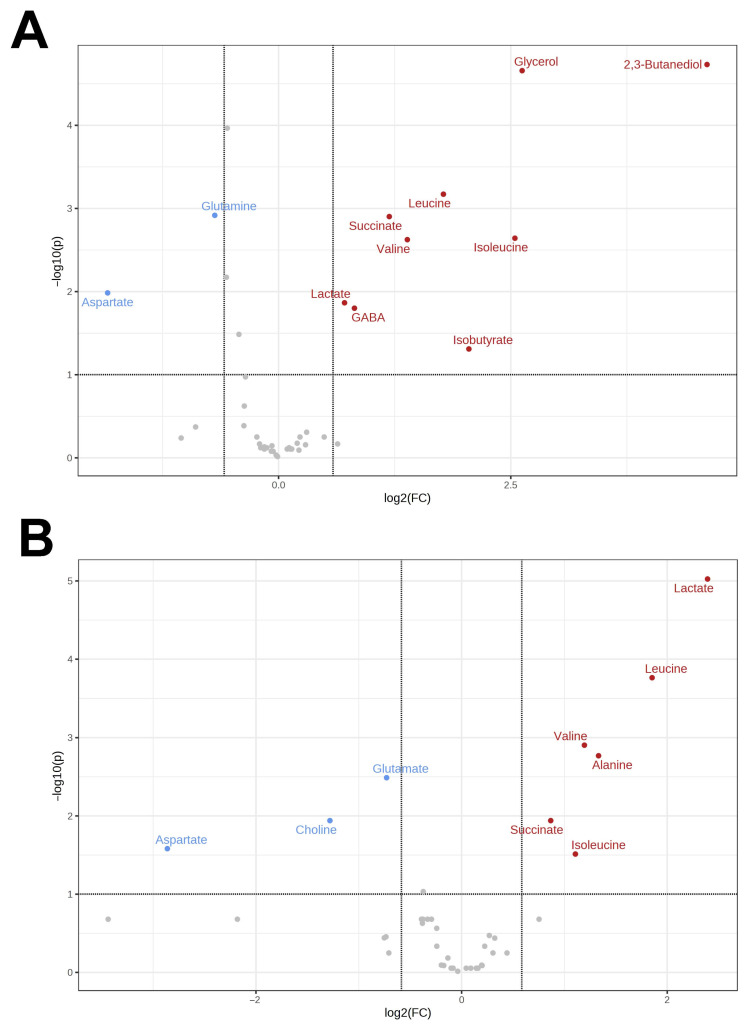
Volcano plots for the comparison of the metabolomic changes in the brain under hypoxia as compared to normoxia for *R. amurensis* (**A**) and *R. dybowskii* (**B**). Substances with increased concentrations under hypoxia are shown in red; decreased, in blue.

A total of 44 substances were identified in the brain of *R. amurensis.* The number of detected metabolites was thus much lower compared to the heart and liver due to the fact that sample (brain) sizes were much smaller and, correspondingly, the limit of detection much higher. The same metabolite set was found in *R. dybowskii*, except for 2,3-butanediol. In the Siberian frog, eight substances increased their concentrations in response to hypoxia, and four, decreased; in the Far Eastern frog, this was six and five, respectively. The list of affected substances included amino acids (alanine, aspartate, glutamate, glutamine, isoleucine, leucine, valine), energy molecules (lactate and succinate), neuromediators (GABA), and osmolytes (phosphocholine). In *R. amurensis*, we also observed an increase in glycerol and 2,3-butanediol. Volcano plots for the comparison of the two species are shown in Figure 3.

When comparing control samples of the two species, significant differences were detected for six molecules: isoleucine, myo- and scyllo-inositol, phosphoethanolamine, putrescine, and taurine. For the individuals exposed to hypoxia, the differences were more pronounced: 18 substances demonstrated statistically significant differences between *R. amurensis* and *R. dybowskii*. This set included the important energy molecules (glucose, lactate, succinate), neuromediators (GABA), and osmolytes (taurine, phosphocholine).

## 4. Discussion

### 4.1. Metabolomic Changes in the Far Eastern and Siberian Frogs in Response to Hypoxia

In this study, we hypothesized that there could be metabolomic differences between the tolerant and the susceptible species exposed to hypoxia. Before the analysis we should bear in mind that the Far Eastern frog was exposed only to brief hypoxia for several hours, up to the limits of its tolerance. Therefore, on the one hand, the Far Eastern frog presumably did not have time to accumulate as many substances as the organs of the Siberian frog. On the other hand, the impact of hypoxia on *R. dybowskii* is much more severe. Based on our findings, we could discern several points in the obtained data.

**Figure 4:**
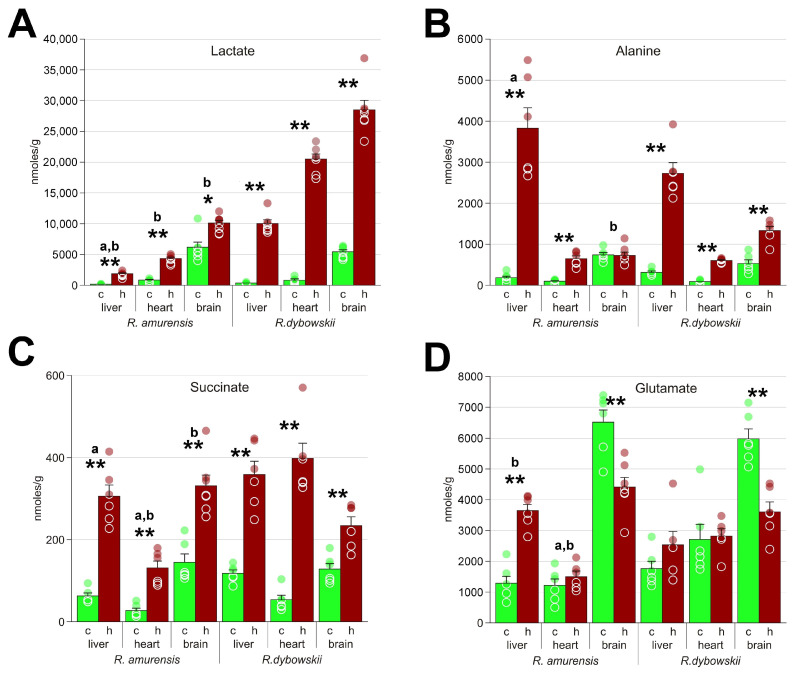
The major molecules associated with energy metabolism in *R. amurensis* and *R. dybowskii* exposed to hypoxia. Green columns (c), normoxia; red (h), hypoxia; a and b above bars indicate statistical significance (Mann–Whitney test *p* < 0.05) of the differences between the two species: a, control samples; b, hypoxia. *, statistical significance of the differences between the control and hypoxic samples. Mann–Whitney test *p* < 0.05; ** *p* < 0.01; circles, individual data points; bar, SEM. (**A**) lactate; (**B**) alanine; (**C**) succinate; (**D**) glutamate.

Despite much shorter exposure duration, all studied organs of the Far Eastern frog had higher concentrations of lactate: about three times more in the brain and five times in the heart and liver (Figure 4A). Lactate is a major damaging factor under hypoxia [18], so the ability of the Siberian frog to deal with it must be the vital factor in adaptation to hypoxia. There are different ways how this might be achieved, including lactate excretion, lowering metabolic rate, or using alternative metabolic pathways.

We found that the levels of succinate in the liver and brain of *R. amurensis* and *R. dybowskii* were similar, while those in the heart were much higher in the latter (Figure 4C). Theoretically, succinate accumulation does not seem to be a factor for *R. dybowskii* survival: the impact of this substance should demonstrate itself only during reoxygenation, while the hypoxia step appears to be the most problematic for the Far Eastern frog. Sometimes we observed that some of the frogs that were considered dead from hypoxia could revive after return to air. However, lower succinate content in the heart of the Siberian frog could be an adaptation for alleviating the reoxygenation stress.

**Figure 5:**
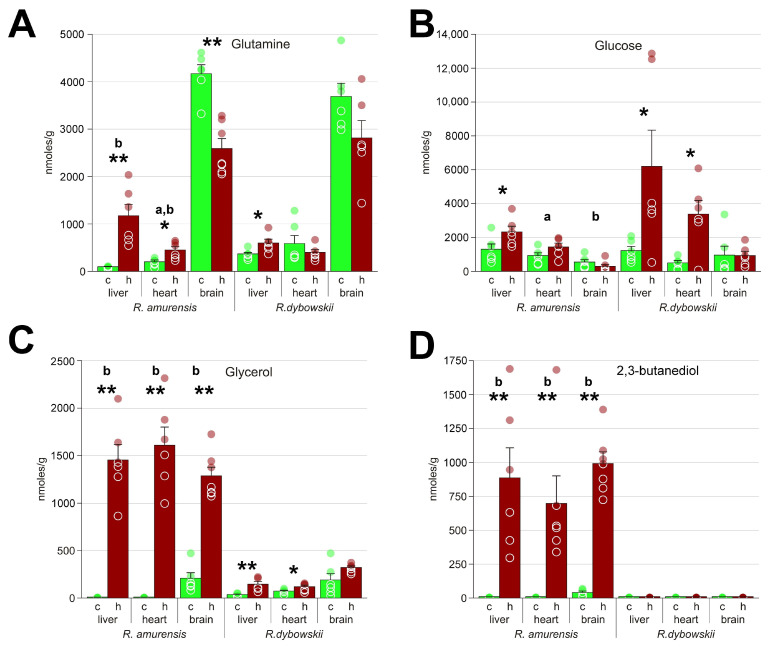
Molecules associated with energy metabolism in *R. amurensis* and *R. dybowskii* exposed to hypoxia. Green columns (c), normoxia; red (h), hypoxia; a and b above bars indicate statistical significance (Mann–Whitney test *p* < 0.05) of the differences between the two species: a, control samples; b, hypoxia. *, statistical significance of the differences between the control and hypoxic samples. Mann–Whitney test *p* < 0.05; ** *p* < 0.01; circles, individual data points; bar, SEM. (**A**) glutamine; (**B**) glucose; (**C**) glycerol; (**D**) 2,3-butanediol.

In an earlier paper, we noted two products whose origin and role were unknown, glycerol and 2,3-butanediol [15]. We found that the latter could not be detected (was either absent or at trace levels) in the control *R. amurensis*, as well as in *R. dybowskii*, but was in relatively high concentrations in all three tissues of the hypoxic Siberian frog (Figure 5C,D). Glycerol was found in the Far Eastern frog and its average content increased in all three organs, but its levels under hypoxia were much lower compared to the Siberian frog, from 4-fold in the brain to 15-fold in the heart.

It is known that glycolysis is much less energy efficient compared to oxidative phosphorylation, so energy would run out fast. However, the amounts of glucose in the organs of the Far Eastern frog are on the average two- to four-fold higher compared to *R. amurensis* (Table S1). This is expected because exposure to hypoxia was much shorter for the former species. Therefore, the lack of energy does not seem to be the main factor causing the death of *R. dybowskii*.

**Figure 6:**
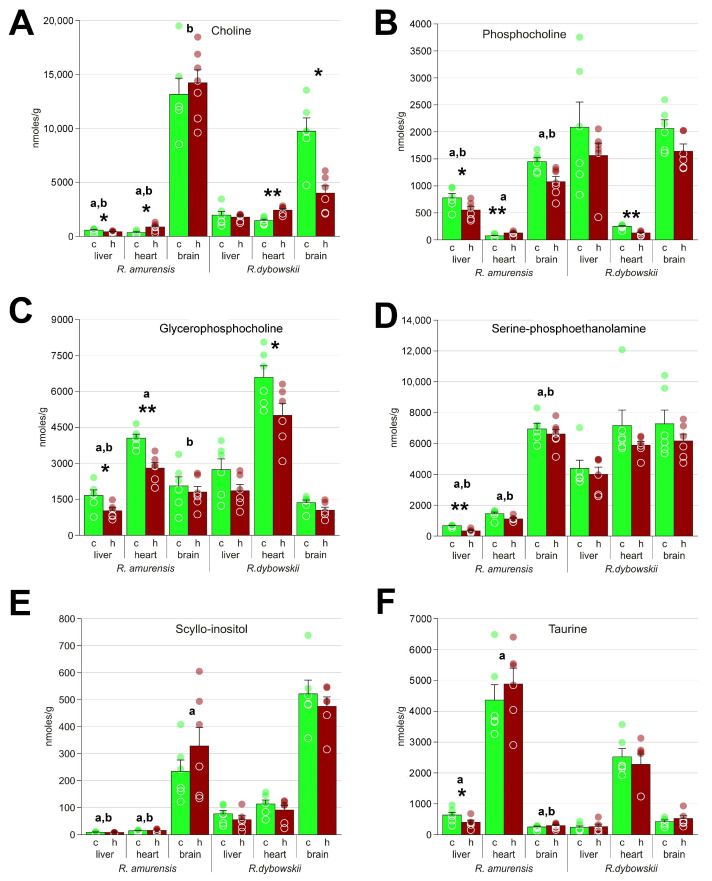
Osmolytes and molecules involved in membrane synthesis in *R. amurensis* and *R. dybowskii* exposed to hypoxia. Green columns (c), normoxia; red (h), hypoxia; a and b above bars indicate statistical significance (Mann–Whitney test *p* < 0.05) of the differences between the two species: a, control samples; b, hypoxia. *, statistical significance of the differences between the control and hypoxic samples. Mann–Whitney test *p* < 0.05; ** *p* < 0.01; circles, individual data points; bar, SEM. (**A**) choline; (**B**) phosphocholine; (**C**) glycerophosphocholine; (**D**) serine-phosphoethnolamine; (**E**) scyllo-inositol; (**F**) taurine.

Among other molecules, we should note the subunits and precursors of membranes choline, phosphocholine, glycerophosphocholine, and serine-phosphoethanolamine (Figure 6A–D) that also have a multitude of other functions. Changes in the concentrations of these molecules are observed in both species in response to hypoxia, suggesting membrane remodeling. On Figure 6A–D, one can see that the baseline content of these substances differs in the two frog species, while the changes under hypoxia usually proceed in the same direction. It might be noteworthy that the Siberian frog has more choline under normoxia compared to the Far Eastern frog, and does not show its decrease under low oxygen, in contrast to the latter species. Choline is the precursor to the neurotransmitter acetylcholine, so the ability to retain its levels might play a role in survival. Glycerophosphocholine is also an osmolyte [19], as well as scyllo-inositol and taurine [20], and these substances also demonstrate significant differences between the two species (Figure 6E,F).

### 4.2. Response of Energy Pathways to Hypoxia in the Siberian Frog

**Figure 7:**
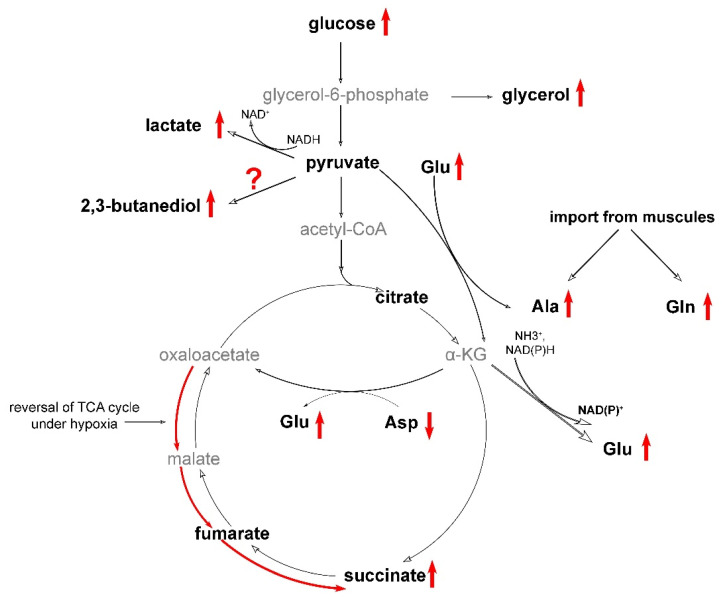
Proposed pathway rearrangements in the liver of *R. amurensis* under hypoxia. Substances found in this study are shown in bold; arrows indicate increased (up) or decreased (down) concentrations; ?, precise reactions unknown. Intermediates that were not detected are shown in gray.

Based on the results obtained in our study, we constructed a diagram (Figure 7) that represents the putative metabolic changes that occur as a result of hypoxia. The cessation of oxidative phosphorylation leads to a decrease in energy production, which can be compensated by glycogenolysis. This was shown, e.g., in the hypoxia-tolerant painted turtle [21,22]. Glycogen is stored predominantly in the liver and the formed glucose is transported to other organs. Our observations support this, as we observed an increase in glucose concentrations in response to hypoxia (Figure 5B). As is commonly reported in the scientific literature, hypoxia-induced glycolysis in vertebrates results in the conversion of glucose to lactate. There are other substances considered as glycolysis end products in hypoxia-tolerant animals. Alanine is widely present in this role in fish [23] and diving turtles [24,25], as well as freeze-tolerant frogs [13,26,27]. Some fish species, e.g., the crucian carp, also employ ethanol as an end product [28,29], and invertebrates can produce many exotic molecules [20].

Lactate is the primary substance that accumulates in hypoxic tissues, as shown in Figure 4A. However, it is toxic in high concentrations since it leads to acidification [30,31]. Therefore, different hypoxia-tolerant organisms demonstrate certain adaptations, like lactate sequestration in turtle shells and bones [30,32] and conversion of pyruvate to ethanol with subsequent excretion in fish [33,34].

Here we also observed the diversion of pyruvate to other metabolic pathways. We detected significant accumulation of alanine in the heart and liver under hypoxic conditions (Figure 4B), indicating its potential role in energy metabolism. However, there is an alternative possibility that the accumulation of alanine in liver might also be the result of amino acid transport from the muscles. This can be hypothesized based on the observed accumulation of glutamine in the liver of the Siberian frog in high quantities (Figure 5A). The usual pathway for the formation of this compound is from glutamate, but this reaction consumes ATP, and, thus, would be unfavorable under hypoxic. On the other hand, glutamine and alanine are imported into liver in the amino acid transport pathways [35]. To obtain energy, they have to be oxidized via the Krebs pathway, but this is impossible under hypoxia.

While ethanol is known to be an alternative end product in fish, it is not typically observed in tetrapods. In a previous study [15], we detected low levels of ethanol in *R. amurensis*, but its volatility makes it sensitive to sample storage and processing.

In addition, two products were also found to be strongly accumulated under hypoxia, glycerol and 2,3-butanediol (Figure 5C,D). Glycerol is known to be formed under hypoxia in yeast [36,37], but we found no similar studies in vertebrates. This process consumes ATP rather than forms it in contrast to glycolysis. However, it regenerates NAD+ [38]. The role and biosynthesis pathways for 2,3-butanediol are unclear, although it is known to be formed in vertebrates [39,40], in some cases in response to hypoxia [41]. 2,3-Butanediol is known to be synthesized from pyruvate in bacteria in several steps, also regenerating NAD+ [42].

As previously mentioned, under hypoxic conditions, alanine is believed to be produced as an alternative product from pyruvate [27]. This reaction is catalyzed by alanine transaminase, which converts pyruvate and glutamate into alanine and α-ketoglutarate. Normally, α-ketoglutarate participates in the Krebs cycle and is converted to succinate via succinyl-CoA. However, under hypoxic conditions, other reactions are expected to occur. Specifically, α-ketoglutarate is expected to be converted to oxaloacetate through a transamination reaction that converts aspartate to glutamate [43]. This is supported by the fact that in Siberian frogs, all detected amino acids, except aspartate, significantly increased in concentration, while the latter was almost depleted.

The currently accepted view is that oxaloacetate is further converted to succinate via the reversal of the Krebs cycle [43,44,45]. Succinate is accumulated under hypoxia in organs of many mammals, including mice and humans [25,44,45], as well as in some hypoxia-tolerant vertebrates, such as the crucian carp [23], and, to a lesser extent, in the diving turtle [24,25,46]. Succinate is considered as one of the main factors contributing to damage under reoxygenation: it is rapidly oxidized, partly leading to reverse electron transport at the mitochondrial complex I, producing free radicals [47,48]. Therefore, one possible pathway to divert from this route could be the formation of glutamate. This process also generates a molecule of NAD+ or NADP+ (Figure 7).

## 5. Conclusions

Our comparison of the metabolomes of the two frog species with contrasting tolerance to hypoxia suggests that the major difference between the two species lies in the ability to lower lactate output. This is achieved by redirecting metabolic pathways towards other products.

## Data Availability

Raw NMR spectra, description of specimens and samples, metabolite concentrations, and the preliminary metabolomic analysis are available at our Animal Metabolite Database repository, experiment ID 109 and 282 (https://amdb.online/amdb/experiments/list) (accessed on 8 August 2023). All other data are available from the corresponding author upon request.

## Supplementary Materials

The following supporting information can be downloaded at: https://www.mdpi.com/article/10.3390/ani13213349/s1, Figure S1: PCA scores plots for the comparison of the liver (A), heart (B), and brain (C) metabolomes of *R. dybowskii* and *R. amurensis* under normoxia; Table S1: Metabolite concentration (in ng/moles) in hypoxic and control frog samples. RD, *R. dybowskii*; RA, *R. amurensis*. ND, not determined (near-zero concentration); NA, not available (absent).

## References

[1] Larson J., Park T.J. (2009). Extreme Hypoxia Tolerance of Naked Mole-Rat Brain. Neuroreport.

[2] Ivy C.M., Sprenger R.J., Bennett N.C., Jaarsveld B., Hart D.W., Kirby A.M., Yaghoubi D., Storey K.B., Milsom W.K., Pamenter M.E. (2020). The Hypoxia Tolerance of Eight Related African Mole-rat Species Rivals That of Naked Mole-rats, despite Divergent Ventilatory and Metabolic Strategies in Severe Hypoxia. Acta Physiol..

[3] Bickler P.E., Buck L.T. (2007). Hypoxia Tolerance in Reptiles, Amphibians, and Fishes: Life with Variable Oxygen Availability. Annu. Rev. Physiol..

[4] Ultsch G.R. (1985). The Viability of Nearctic Freshwater Turtles Submerged in Anoxia and Normoxia at 3 and 10 °C. Comp. Biochem. Physiol. A.

[5] Ultsch G.R. (1989). Ecology and Physiology of Hibernation and Overwintering among Freshwater Fishes, Turtles, And Snakes. Biol. Rev..

[6] Reese S.A., Jackson D.C., Ultsch G.R. (2002). The Physiology of Overwintering in a Turtle That Occupies Multiple Habitats, the Common Snapping Turtle (*Chelydra serpentina*). Physiol. Biochem. Zool..

[7] Berman D.I., Bulakhova N.A., Meshcheryakova E.N. (2019). The Siberian Wood Frog Survives for Months Underwater without Oxygen. Sci. Rep..

[8] Bulakhova N.A., Meshcheryakova E.N., Berman D.I. (2023). Pallas’ Spadefoot Pelobates vespertinus (Pelobatidae, Amphibia) Tolerates Extreme Hypoxia. Eur. Zool. J..

[9] Wishart D.S. (2019). NMR Metabolomics: A Look Ahead. J. Magn. Reson..

[10] Zelentsova E.A., Yanshole L.V., Tsentalovich Y.P., Sharshov K.A., Yanshole V.V. (2022). The Application of Quantitative Metabolomics for the Taxonomic Differentiation of Birds. Biology.

[11] Niu Y., Cao W., Wang J., He J., Storey K.B., Ding L., Tang X., Chen Q. (2021). Freeze Tolerance and the Underlying Metabolite Responses in the Xizang Plateau Frog, Nanorana parkeri. J. Comp. Physiol. B.

[12] Niu Y., Zhang X., Men S., Storey K.B., Chen Q. (2023). Integrated Analysis of Transcriptome and Metabolome Data Reveals Insights for Molecular Mechanisms in Overwintering Tibetan Frogs, Nanorana parkeri. Front. Physiol..

[13] Shekhovtsov S.V., Bulakhova N.A., Tsentalovich Y.P., Zelentsova E.A., Meshcheryakova E.N., Poluboyarova T.V., Berman D.I. (2022). Metabolomic Analysis Reveals That the Moor Frog Rana arvalis Uses Both Glucose and Glycerol as Cryoprotectants. Animals.

[14] Shekhovtsov S.V., Bulakhova N.A., Tsentalovich Y.P., Zelentsova E.A., Meshcheryakova E.N., Poluboyarova T.V., Berman D.I. (2021). Biochemical Response to Freezing in the Siberian Salamander Salamandrella keyserlingii. Biology.

[15] Shekhovtsov S.V., Bulakhova N.A., Tsentalovich Y.P., Zelentsova E.A., Yanshole L.V., Meshcheryakova E.N., Berman D.I. (2020). Metabolic Response of the Siberian Wood Frog Rana amurensis to Extreme Hypoxia. Sci. Rep..

[16] Bulakhova N.A., Mazanaeva L.F., Mescheryakova E.N., Berman D.I. (2020). Resistance of Iranian Long-Legged Wood Frog *Rana macrocnemis* (Amphibia, Anura) to Negative Temperatures on Land and to Hypoxia in Water during Overwintering. Herpetol. Notes.

[17] Tsentalovich Y.P., Yanshole V.V., Yanshole L.V., Zelentsova E.A., Melnikov A.D., Sagdeev R.Z. (2019). Seasonal Variations and Interspecific Differences in Metabolomes of Freshwater Fish Tissues: Quantitative Metabolomic Profiles of Lenses and Gills. Metabolites.

[18] Jackson D.C. (2000). Living without Oxygen: Lessons from the Freshwater Turtle. Comp. Biochem. Physiol. A.

[19] Gallazzini M., Burg M.B. (2009). What’s New About Osmotic Regulation of Glycerophosphocholine. Physiology.

[20] Hochachka P.W., Somero G.N. (2002). Biochemical Adaptation: Mechanism and Process in Physiological Evolution.

[21] Clark V.M., Miller A.T. (1973). Studies on Anaerobic Metabolism in the Fresh-Water Turtle (*Pseudemys scripta elegans*). Comp. Biochem. Physiol. A.

[22] Pamenter M.E., Richards M.D., Buck L.T. (2007). Anoxia-Induced Changes in Reactive Oxygen Species and Cyclic Nucleotides in the Painted Turtle. J. Comp. Physiol. B.

[23] Dahl H.-A., Johansen A., Nilsson G.E., Lefevre S. (2021). The Metabolomic Response of Crucian Carp (*Carassius carassius*) to Anoxia and Reoxygenation Differs between Tissues and Hints at Uncharacterized Survival Strategies. Metabolites.

[24] Buck L. (2000). Succinate and Alanine as Anaerobic End-Products in the Diving Turtle (*Chrysemys picta bellii*). Comp. Biochem. Physiol. B.

[25] Bundgaard A., James A.M., Gruszczyk A.V., Martin J., Murphy M.P., Fago A. (2019). Metabolic Adaptations during Extreme Anoxia in the Turtle Heart and Their Implications for Ischemia-Reperfusion Injury. Sci. Rep..

[26] Storey K.B. (1987). Organ-Specific Metabolism during Freezing and Thawing in a Freeze-Tolerant Frog. Am. J. Physiol. Integr. Comp. Physiol..

[27] Storey K.B., Storey J.M. (2004). Metabolic Rate Depression in Animals: Transcriptional and Translational Controls. Biol. Rev..

[28] Vornanen M., Stecyk J.A.W., Nilsson G.E. (2009). Chapter 9 The Anoxia-Tolerant Crucian Carp (*Carassius carassius* L.). Functional Metabolism: Regulation and Adaptation.

[29] Johnston I.A., Bernard L.M. (1983). Utilization of the Ethanol Pathway in Carp Following Exposure to Anoxia. J. Exp. Biol..

[30] Jackson D. (1997). Lactate Accumulation in the Shell of the Turtle *Chrysemys picta bellii* during Anoxia at 3 °C and 10 °C. J. Exp. Biol..

[31] Kraut J.A., Madias N.E. (2014). Lactic Acidosis. N. Engl. J. Med..

[32] Davis E.C., Jackson D.C. (2007). Lactate Uptake by Skeletal Bone in Anoxic Turtles, Trachemys scripta. Comp. Biochem. Physiol. A.

[33] Nilsson G.E. (1988). A Comparative Study of Aldehyde Dehydrogenase and Alcohol Dehydrogenase Activities in Crucian Carp and Three Other Vertebrates: Apparent Adaptations to Ethanol Production. J. Comp. Physiol. B.

[34] Dhillon R.S., Mandic M., Yao L., Cao Z.-D., Fu S.-J., Brauner C.J., Wang Y.S., Richards J.G. (2018). Ethanol Metabolism Varies with Hypoxia Tolerance in Ten Cyprinid Species. J. Comp. Physiol. B.

[35] Paulusma C.C., Lamers W.H., Broer S., van de Graaf S.F.J. (2022). Amino Acid Metabolism, Transport and Signalling in the Liver Revisited. Biochem. Pharmacol..

[36] Nordström K. (1966). Yeast Growth and Glycerol Formation. Acta Chem. Scand..

[37] Ansell R., Granath K., Hohmann S., Thevelein J.M., Adler L. (1997). The two isoenzymes for yeast NAD+-dependent glycerol 3-phosphate dehydrogenase encoded by GPD1 and GPD2 Have Distinct Roles in Osmoadaptation and Redox Regulation. EMBO J..

[38] Chen Z., Liu D. (2016). Toward Glycerol Biorefinery: Metabolic Engineering for the Production of Biofuels and Chemicals from Glycerol. Biotechnol. Biofuels.

[39] Casazza J.P., Frietas J., Stambuk D., Morgan M.Y., Veech R.L. (1987). The Measurement of 1, 2-Propanediol, D, L-2, 3-Butanediol and Meso-2, 3-Butanediol in Controls and Alcoholic Cirrhotics. Alcohol Alcohol Suppl..

[40] Montgomery J.A., Jetté M., Brunengraber H. (1990). Assay of Physiological Levels of 2, 3-Butanediol Diastereomers in Blood and Urine by Gas Chromatography-Mass Spectrometry. Anal. Biochem..

[41] Heer K.R., Althaus U., Mettler D., Schilt W., Thoelen H. (1991). 2,3-Butanediol in Experimental Myocardial Ischaemia in Pigs. Eur. Heart J..

[42] Ji X.-J., Huang H., Ouyang P.-K. (2011). Microbial 2,3-Butanediol Production: A State-of-the-Art Review. Biotechnol. Adv..

[43] Fago A. (2022). New Insights into Survival Strategies to Oxygen Deprivation in Anoxia-tolerant Vertebrates. Acta Physiol..

[44] Chouchani E.T., Pell V.R., Gaude E., Aksentijević D., Sundier S.Y., Robb E.L., Logan A., Nadtochiy S.M., Ord E.N.J., Smith A.C. (2014). Ischaemic Accumulation of Succinate Controls Reperfusion Injury through Mitochondrial ROS. Nature.

[45] Chinopoulos C. (2019). Succinate in Ischemia: Where Does It Come From?. Int. J. Biochem. Cell Biol..

[46] Bundgaard A., Ruhr I.M., Fago A., Galli G.L.J. (2020). Metabolic Adaptations to Anoxia and Reoxygenation: New Lessons from Freshwater Turtles and Crucian Carp. Curr. Opin. Endocr. Metab. Res..

[47] Chouchani E.T., Pell V.R., James A.M., Work L.M., Saeb-Parsy K., Frezza C., Krieg T., Murphy M.P. (2016). A Unifying Mechanism for Mitochondrial Superoxide Production during Ischemia-Reperfusion Injury. Cell Metab..

[48] Kula-Alwar D., Prag H.A., Krieg T. (2019). Targeting Succinate Metabolism in Ischemia/Reperfusion Injury. Circulation.

