# Supplementary material for: Metabolomic Profiling Reveals Differences in Hypoxia Response between Far Eastern and Siberian Frogs
Source: Animals (Basel). 2023 Oct 27;13(21):3349. doi: 10.3390/ani13213349 (PMC10647746; doi:10.3390/ani13213349)
Supplement: Supplementary file 1 [file animals-13-03349-s001.zip › Figure S1.pdf]

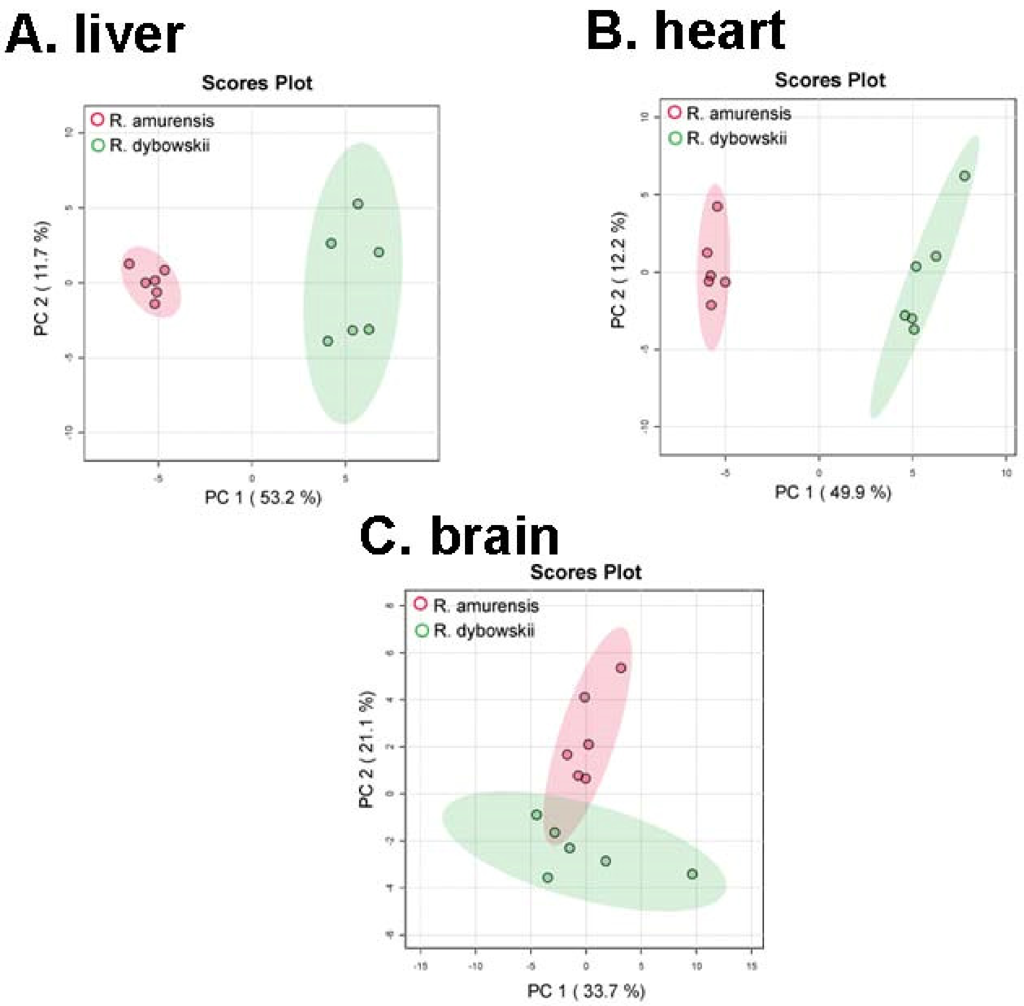

Figure S1. PCA scores plots for the comparison of the liver (A), heart (B), and brain (C) metabolomes of *R. dybowskii* and *R. amurensis* under normoxia.
